# Supplementary material for: Proteotranscriptomic Profiling of the Toxic Mucus of Kulikovia alborostrata (Pilidiophora, Nemertea)
Source: Toxins (Basel). 2024 Dec 26;17(1):5. doi: 10.3390/toxins17010005 (PMC11768458; doi:10.3390/toxins17010005)
Supplement: Supplementary file 1 [file toxins-17-00005-s001.zip › Supplementary File S4.zip!/ORF 076246 LINALB/5646dc0998f06131/hit_report.pdf]

Email	vas9i-kz@mail.ru
Description	putative_toxin_cistein_rich_
Date	Wed Apr 5 07:02:50 BST 2023
Unique Job ID	5646dc0998f06131

Detailed template information

#	Template	Alignment Coverage	3D Model	Confidence	% i.d.	Template Information
1	c6enaA_	Alignment	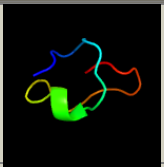	94.4	52	**PDB header:**toxin<br>**Chain:** A: **PDB Molecule:**nemertide alpha-1;<br>**PDBTitle:** nemertide alpha-1<br>**PDB Entry:** PDBe RCSB PDBj
2	d1h5oa_	Alignment	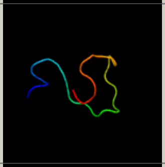	22.4	38	**Fold:** Defensin-like<br>**Superfamily:** Defensin-like<br>**Family:** Myotoxin<br>**PDB entry:** PDBe RCSB PDBj
3	c1z99A_	Alignment	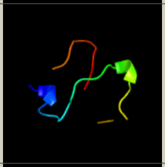	18.4	38	**PDB header:**toxin<br>**Chain:** A: **PDB Molecule:**crotamine;<br>**PDBTitle:** solution structure of crotamine, a myotoxin from crotalus durissus2 terrificus<br>**PDB Entry:** PDBe RCSB PDBj
4	d1vtxa_	Alignment	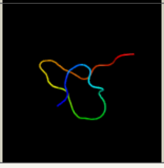	44.8	42	**Fold:** Knottins (small inhibitors, toxins, lectins)<br>**Superfamily:** omega toxin-like<br>**Family:** Spider toxins<br>**PDB entry:** PDBe RCSB PDBj
